# Fasting Enhances the Acute Toxicity of Acrylonitrile in Mice via Induction of CYP2E1

**DOI:** 10.3390/toxics10060337

**Published:** 2022-06-19

**Authors:** Suhua Wang, Guangwei Xing, Fang Li, Bobo Yang, Yu Zhang, Michael Aschner, Rongzhu Lu

**Affiliations:** 1 Department of Preventive Medicine and Public Health Laboratory Science, School of Medicine, Jiangsu University, 301 Xuefu Road, Zhenjiang 212013, China; wsh_9321@163.com (S.W.); xgw_123@163.com (G.X.); lfsjy@ujs.edu.cn (F.L.); hkzx93@126.com (B.Y.); zyyy1018@163.com (Y.Z.); 2 Department of Molecular Pharmacology, Albert Einstein College of Medicine, Bronx, NY 10461, USA; michael.aschner@einsteinmed.edu; 3 Center for Experimental Research, Affiliated Kunshan Hospital to Jiangsu University School of Medicine, Kunshan, Suzhou 215300, China

**Keywords:** fasting, acrylonitrile, CYP2E1, acute toxicity, glutathione

## Abstract

Cytochrome P450 2E1 (CYP2E1) plays an essential role in the susceptibility to acute acrylonitrile (AN)-induced toxicity. Here, we investigated the toxicity and mechanism of AN in fasting mice and potential underlying mechanisms. Convulsions, loss of righting reflex, and death 4 h after AN treatment were observed and recorded for each group of mice. Relative to ad lib-fed mice, 48 h fasting significantly increased the acute toxicity of AN, as noted by a more rapid onset of convulsions and death. In addition, fasting significantly enhanced CYP2E1-mediated oxidative metabolism of AN, resulting in increased formation of CN^-^ (one of the end-metabolites of AN). Moreover, fasting decreased hepatic GSH content, abrogating the detoxification of GSH. However, trans-1,2-dichloroethylene (DCE), a CYP2E1 inhibitor, altered the level of hepatic CYP2E1 activity in response to fasting, reduced the acute toxic symptoms of AN and the content of CN^-^ in AN-treated mice. These data establish that fasting predisposes to AN toxicity, attributable to induced CYP2E1 and reduced hepatic GSH.

## 1. Introduction

Though health benefits of fasting on aging, antioxidant stress, metabolism, and cardiovascular disease have been confirmed in human and animal studies [1,2,3], fasting or/and nutrient deficiency can also alter the metabolism of chemicals by regulating the contents and activity of relevant metabolic enzymes, which, in turn, may affect the in vivo fate of some exogenous chemicals, and thus influence their therapeutic efficacy and/or toxicity [4,5,6]. In fact, undernutrition is a growing global problem that is exacerbated by global warming, the COVID-19 pandemic, and wars leading to reduced crop production and export to food-reliant populations and forced migration. Under these and other circumstances, individuals may be hungry or consume insufficient food for days/weeks/months, resulting in loss of body weight and altered metabolic activity that may lead to illness and death. However, the regulation of chemical exposure is often based on laboratory studies of animals that are fed and watered ad libitum. Studies to examine changes in the response of animals to test chemicals as a function of undernourishment are almost never carried out, in part because of ethical concerns but also because undernourished subjects may be of limited direct concern to industrialized societies.

Therefore, undernutrition must be corroborated in the assessment of risk for certain chemical-induced neurological diseases, and in some cases, undernutrition is a prerequisite for neurotoxic manifestation [7].

Acrylonitrile (AN), an important industrial and environmental chemical, is widely used in the production of acrylic fibers, plastics, synthetic rubber, and acrylamide. Though routes of human exposure to AN include inhalation from occupational exposure, car exhaust, cigarette, and fire smoke as well as oral exposures from drinking water and food products [8,9,10], oral exposures represent a very minor overall contributor to AN exposures, with inhalation representing the dominant exposure route for occupational and general population human exposures, according to the Agency for Toxic Substances and Disease Registry (ATSDR) [11].

AN toxicity is mediated, at least in part, by its metabolism by cytochrome P450 2E1 (CYP2E1) into CN^-^ [12], which is responsible for much of the acute toxicity of AN. Oxidative damage is inherent to AN exposure, given its ability to deplete intracellular GSH levels [13,14]. CYP2E1 is a major constitutive enzyme of the mammalian liver, the activity of which is enhanced by fasting [15,16].

**Figure 1:**
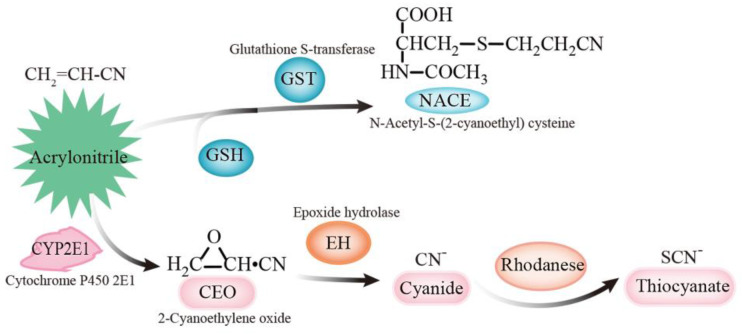
The metabolic pathways of acrylonitrile.

AN is metabolized via two different routes [17,18]. One route of AN metabolism is its conversion to cyanide via a cytochrome P450-dependent mixed-function oxidase system. *In vitro* experiments confirmed that this biotransformation proceeds via glycidonitrile (catalyzed by hepatic monooxygenases) and glycolaldehyde cyanohydrin (catalyzed by epoxide hydrase) [19]. Cyanohydrin is spontaneously decomposed to cyanide, which is then metabolized in vivo to thiocyanate by rhodanese. The other route of AN-metabolism, however, proceeds via its conjugation with glutathione catalyzed by glutathione S-alkenetransferases to N-acetyl-S-(2-cyanoethyl) cysteine as a final product. The glutathione-dependent pathway is considered a detoxication route, whilst the oxidative pathway leads to a genotoxic epoxide, cyanoethylene oxide, and to the production of cyanide (Figure 1). Some, but not all, of the toxicity of AN may be due to the release of cyanide, which, in turn, inhibits numerous enzymes, including cytochrome oxidase, resulting in cellular asphyxiation. Toxicity not related to cyanide formation is due to the formation of reactive vinyl groups and epoxide intermediates which can deplete glutathione stores and cause liver damage [11].

Given the above, the present study investigated the effects of fasting on AN-induced acute toxicity and the underlying mechanisms. It provides a theoretical basis for a further comprehensive study of the health effects of fasting and individualized protective countermeasures against chemicals, whether of synthetic or natural origin.

## 2. Materials and Methods

### 2.1. Chemicals

AN (99% purity) was obtained from the Sinopect Shanghai Petrochemical Company (Jinshan District, Shanghai, China). Trans-1, 2-dichloroethylene, *p*-nitrophenol, and *p*-nitrocatechol were purchased from Sigma Chemical Co (Shanghai, China). The glutathione (GSH) and protein determination kits were purchased from Nanjing Jiancheng Bioengineering Institute (Nanjing, Jiangsu, China). Other commonly used chemical reagents of high purity were of domestic or imported origin.

### 2.2. Animals and Treatment

Male Kunming mice (4–5 weeks old, 8 mice per group) with a body weight of 20 ± 2 g (SYXK (SU) 2018-0035]) were provided by the Laboratory Animal Center of Jiangsu University. After one week of normal adaptive feeding, the mice were randomly divided into two groups. Animals in the fasting group received no food for a period of 48 h prior to treatment with test agents. Whereas drinking water was provided ad libitum throughout the adaptive feeding and fasting periods of the fasting group, mice in the normal group (controls) were continuously provided with food and water ad libitum. At the end of the fasting period, animals in both groups were individually weighed, injected intraperitoneally (i.p. AN (10, 20, or 40 mg/kg), and trans-1,2-dichloroethylene (DCE, 100 mg/kg). DCE, a CYP2E1 inhibitor, was given 2 h before AN. AN was dissolved in a vehicle (normal saline), and mice in control groups of fasted and non-fasted were treated with an equivalent volume of vehicle at times matching the injection of AN. All animal study protocol regarding animal care and welfare was checked and approved by the Institutional Animal Care and Use Committee of Jiangsu University (No. UJS-IACUC-AP-202004001).

### 2.3. Behavioral Observations

Immediately after AN or vehicle challenge injection, the animals were individually placed in cages and observed for abnormal signs, namely convulsions, the righting reflex, and death. We recorded the time to onset of convulsions, the time to loss of righting reflex, and the time to death, within 4 h after AN treatment.

### 2.4. Sample Preparation

Mice treated with 40 mg/kg AN or vehicle were decapitated 30 min after AN administration. The liver and brain were dissected, dried on filter paper, weighed, and stored at −80 °C for the detection of CYP2E1 activity, CN^-^ and GSH content.

### 2.5. Preparation of Liver Microsomes

Liver homogenates (10%) were prepared in chilled potassium phosphate (5 mM; pH 7.4) and then centrifuged at 11,800× *g* for 20 min at 4 °C. The supernatants (1.5 mL) were added to ice-cold CaCl_2_ (final concentration, 8 mM CaCl_2_) for 30 min and then re-centrifuged at 11,800× *g* for 20 min. The resulting microsomal pellets were resuspended in 100 μL of 0.1 M sodium phosphate (pH 7.4) containing 2 mM MgCl_2_ and stored at −80 °C until analysis.

### 2.6. CYP2E1 Activity, CN^-^ Content, and GSH Assay

CYP2E1 activity was assessed on hepatic microsomes and based on the principle of the rate of oxidation of *p*-nitrophenol to *p*-nitrocatechol in the presence of NADPH [20,21]; results were expressed as nmol/min/mg of microsomal protein. CN^-^ concentrations in brain and liver homogenates were determined by the Conway microdiffusion method [22], as previously described [23]; results were expressed as μgCN^-^/g tissue. GSH in the deproteinized supernatant was estimated by using the GSH assay kit according to the manufacturer’s instruction; results were expressed as nmol GSH/mg protein.

### 2.7. Statistical and Data Analysis

Data are expressed as the mean ± standard deviation (SD) of eight mice. Behavioral and mortality data were analyzed by Fisher’s exact test, while other data were analyzed by one-way analysis of variance (ANOVA). The Student–Newman–Keul’s test was applied to determine the significance of differences in means between groups. A *p* value less than 0.05 was considered statistically significant. All statistical analyses were performed using SPSS for Windows (Version 16.0; SPSS Inc., Chicago, IL, USA).

## 3. Results

### 3.1. Effect of Fasting on Acute Toxicity in Mice Exposed to Different Doses of AN

After 48-hour fasting, the mice’s weight significantly decreased (Table 1). When mice were treated with 20 mg/kg AN, seven animals in the fasting group displayed convulsions, which was significantly higher than that in the AN alone group (*p* < 0.05). When the dose was increased to 40 mg/kg AN, all mice in both groups exhibited convulsive behavior, whereas all 8 fasting mice died within 4 h of treatment with 40 mg/kg AN group, and only one comparably treated animal died during the same period. Fasting shortened the survival time from 129 min to 56 min. Taken together, these data show that fasting increases acute toxicity and shortens the survival time in AN-intoxicated mice.

### 3.2. Effects of Fasting and DCE on Hepatic CYP2E1 Activity

**Figure 2:**
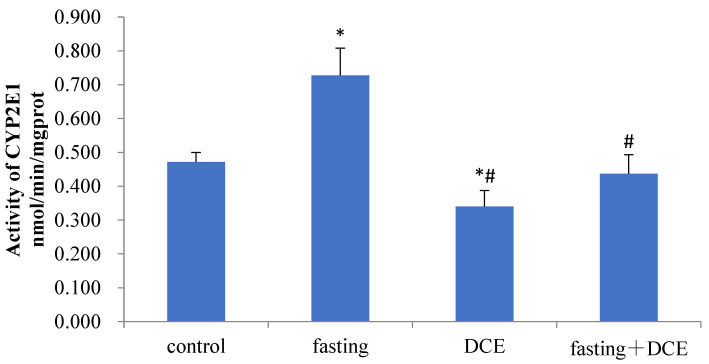
Effect of different treatments on the activity of hepatic CYP2E1 in mice. *: Compared with the control group, *p* < 0.05. #: Compared with fasting group, *p* < 0.05.

CYP2E1 activity was significantly increased in the fasting vs. the control group (*p* < 0.05, Figure 2). CYP2E1 activity in mice treated with DCE alone was significantly lower than enzyme activity in normal control and fasted mice(*p* < 0.05, Figure 2). DCE, a CYP2E1 inhibitor, altered the level of hepatic CYP2E1 activity caused by fasting.

### 3.3. Effects of Fasting and DCE on AN-Induced Acute Toxicity

Animals treated with 40 mg/kg AN were used to assess the effect of DCE on the acute toxicity of AN in fasting mice. The number of mice with loss of righting reflex and death in the fasting group significantly increased compared with mice treated with AN alone, and the time to loss of righting reflex and death was shortened. The acute toxic signs of AN in animals pretreated with DCE were reduced, as indicated by the number of convulsions and loss of the righting reflex (Table 2). Although the mortality did not significantly decrease, the survival time of fasting mice was prolonged.

### 3.4. Effects of Fasting and DCE on Tissue CN^-^ in Mice Treated with AN

The effects of AN treatment with fasting and DCE on tissue CN^-^ content are summarized in Table 3. Compared with the AN alone group, the mean content of CN^-^ in liver and brain tissue of fasting mice was significantly increased. Pretreatment with DCE reduced the content of CN^-^ in tissue of mice treated with AN. A low concentration of CN^-^ was detected in the liver of mice in the DCE + 40 mg/kg AN group. Although the brain CN^-^ content of mice in the fasting + DCE + 40 mg/kg AN group was not significantly lower than that in mice treated with AN alone, compared with the fasting + 40 mg/kg AN group, the content of CN^-^ in liver and brain significantly decreased (*p* < 0.05).

### 3.5. Effects of Fasting and DCE on Tissue GSH Level in Mice Treated with AN

Compared with the normal control group, fasting and AN treatment significantly reduced hepatic GSH content in mice. This change was not observed in the DCE group. Hepatic GSH levels were significantly lower in the AN-treated rats with fasting and/or DCE pretreatment compared with the AN alone group (*p* < 0.05). The change in GSH content in the brain tissue was distinct from that in liver tissue; a significant difference was observed between the fasting + DCE + 40 mg/kg AN group and the 40 mg/kg AN group (Table 3).

## 4. Discussion

Fasting, as a measure of caloric restriction, has been widely adopted in modern society as a potential intervention for improved physical and mental health, reducing the risk of cardiovascular disease, improving insulin sensitivity in diabetes mellitus, reducing oxidative stress, enhancing cognitive function, as well as cancer treatment [24,25,26,27,28]. For instance, Cheng et al. demonstrated that prolonged fasting protects hematopoietic stem cells (HSC) against chemotherapeutic toxicity and promotes HSC regeneration [26]. While fasting may have beneficial effects, it may also be accompanied by changes in muscle mass and physical activity level that may adversely impact long-term health [29].

It is well known that various dietary factors have marked effects on the metabolism of drugs, environmental chemicals, and certain endogenous substrates [6]. Fasting also affects the in vivo fate of ingested xenobiotics and thus influences their pharmacological activity and/or toxicity. Similarly, fasting may impact the metabolic fate of environmental pollutants and thereby influence their toxic potential [30,31,32,33,34]. The levels and activities of cytochrome P4502E1, the major P450 enzyme involved in the activation of several low molecular weight toxins, carcinogens, and drugs [35], are influenced by a variety of factors, including diet. A wealth of research has shown that fasting can cause induction of CYP2E1 activity in rodents [16, 33, 36,37], thereby resetting the threshold and/or degree of chemical toxicity. For instance, CYP2E1 is the primary mechanism promoting the bioactivation-based liver toxicity of thioacetamide in diet-restricted rats [34]. Another study has shown that fasting significantly enhanced cytochromes P450 enzymes leading to increased pyrrolizidine alkaloid-induced hepatotoxicity [37]. Accordingly, such individuals may be at increased risk of adverse effects caused by the formation of CYP2E1-mediated metabolites of environmental agents. Several studies exist on the effects of fasting on drug metabolism by CYP2E1. According to Tsuchiya et al., fasting or food restriction exacerbated acetaminophen-induced acute liver injury by induction of CYP2E1 and reduction in liver GSH contents [33]. O’Shea et al. noted that the efficacy of CYP2E1 drug substrates is reduced in obese patients [38]. Previous work in our laboratory has shown that enhanced CYP2E1 has a critical role in the outcome of acute AN-induced toxicity [23,39]. The induction of cytochrome CYP2E1 in diabetic rats has been shown to increase the acute toxicity of AN [39]. CYP2E1 inhibitors are effective in prolonging the survival time of rats intoxicated by AN [23], and the protective effect of phenethyl isothiocyanate in rats with high CYP2E1 activity has been shown to be related to its ability to inhibit liver CYP2E1 activity [40].

Given these observations, we studied herein the effects of fasting on CYP2E1 and the acute toxicity of AN in mice. Due to the oxidative metabolism of AN has a much greater impact on humans than on rodents [41], and CYP2E1 activity plays a critical role in human susceptibility to AN [42]. Forty-eight-hour fasting [16,43,44], mouse models with increased expression of CYP2E1 [45] and the CYP2E1 inhibitor, DCE, were adopted in this study. The present study results indicate that when AN is injected intraperitoneally after fasting for 48 h, young adult mice showed distinct signs of cyanide toxicity. The number of animals displaying signs of acute toxicity (convulsion, disappearance of righting reflex, and death) increased, and the survival time was shortened. The increase in CN^-^ concentration in liver and brain tissue shows that fasting enhanced the acute toxicity of AN; pretreatment with DCE reduced the content of CN^-^ in tissues of fasted AN-treated mice. The increased toxicity of AN was related to the activity of CYP2E1, a key metabolic enzyme of AN induced by fasting. Moreover, the results showed that fasting significantly reduced the hepatic concentration of GSH, an observation consistent with the experimental results of Szkudelski and associates [32]. In view of the role of GSH in maintaining the antioxidant levels and that oxidative stress is one of the prevailing toxic mechanisms of AN, exposure to AN under fasting conditions can aggravate GSH depletion, aggravating oxidative stress and the ensuing tissue damage. Our data not only broaden the understanding of AN toxicity but also raise concerns about the risk of exposure to chemicals in populations with restrictive diets. Our findings also establish attenuation of toxicity upon CYP2E1 inhibition. Altogether, these findings suggest that fasting can be a predisposing factor for AN toxicity. Fasting-enhanced acute AN intoxication might involve mainly two pathways, including (1) enhanced CYP2E1-mediated metabolic activation leading to increased formation of reactive metabolite CN^-^, promoting mortality, and (2) reduced hepatic GSH content resulting in impairment of the AN detoxification pathway. Taken together, the diet has a critical impact on human health. Considering the effects of fasting, the present study establishes that the assessment of metabolite-mediated toxins should consider feeding and/or fasting conditions and their contribution to xenobiotic-induced toxicity. In particular, for workers, especially those at high risk of occupational exposure, the diet should be considered as a potential confounding variable in risk assessment.

From the point of public health toxicology, our study does have several limitations. First, the findings in our study do not completely mimic AN exposure representing adverse effects in humans suffering even severe body weight loss. Secondly, the findings may not be credibly extrapolating to even acute accidental/occupational AN exposure risks. Therefore, our findings merely indicate that AN is an additional experimental compound corroborating previously published findings that severe diet restriction may enhance chemical/drug toxicity by fasting-associated increases in CYP2E1 metabolism. Thirdly, the protocol used in our study (extreme fasting stress animal model that is not equivalent to human weight loss; i.p. dosing) does not infer any potential AN human risk potential, and future studies conducted by the more human-relevant inhalation route and using weight loss protocols such a partial diet restriction vs. total feed restriction as used in this study would be necessary to realistically examine any such risk potential.

In summary, the present study as a case demonstrates the important role of severe acute undernourishment and related changes in CYP2E1 enzyme activity on the toxic potential of a representative industrial chemical (AN) in rodents. Given that ten and a growing percentage of the human population experiences acute and chronic undernourishment, this factor should be addressed in animal studies that seek to assess the human toxic potential of industrial and other chemicals.

## 5. Conclusions

Fasting was an important predisposing factor for AN toxicity, likely attributable to enhanced CYP2E1 and reduced hepatic GSH. We suggest that feeding and/or fasting conditions and/or should be considered as a potential confounding variable in the risk assessment of industrial and environmental chemical exposure. In summary, it should be noted that inhibition ofCYP2E1 will be further studied as a promising chemopreventive agent for acute AN intoxication in individuals with elevated CYP2E1 activity.

## Figures and Tables

**Table 1:** Effect of fasting on acute toxicity in mice exposed to different doses of AN.

Group	Weight (g)	Convulsions	Loss of Righting Reflex	Time to Loss of Righting Reflex (min)	Mortality	Time to Mortality (min)
10 mg/kg AN	26.6 ± 1.1	2	0	-	0	-
fasting + 10 mg/kg AN	18.4 ± 0.5 ^a^	3	0	-	0	-
20 mg/kg AN	26.7 ± 2.0	1	2	12.0 ± 4.2	0	-
fasting + 20 mg/kg AN	19.3 ± 1.5 ^a^	7 ^a^	4	13.0 ± 5.7	1	65
40 mg/kg AN	26.9 ± 1.2	8	6	13.5 ± 5.5	1	129
fasting + 40 mg/kg AN	18.3 ± 1.2 ^a^	8	8	13.4 ± 4.6	8 ^a^	56 ± 11.7

^a^: Compared with the same dose group, *p* < 0.05.

**Table 2:** Effects of fasting and DCE on acute toxicity in mice exposed to 40 mg/kg AN.

Group	Weight (g)	Convulsion(n)	Loss of Righting Reflex (n)	Time to Loss of Righting Reflex (min)	Mortality (n)	Time to Death (min)
40 mg/kg AN	28.5 ± 0.9	6	4	30.0 ± 9.8	0	-
DCE + 40 mg/kg AN	29.4 ± 1.4	0 ^a^	0 ^a^	-	0	-
Fasting + 40 mg/kg AN	19.5 ±0.9 ^a^	4	8 ^a,b^	15.8 ± 5.1	5 ^a^	62.6 ± 18.3
Fasting + DCE + 40 mg/kg AN	19.9 ± 0.7 ^a,b^	0 ^ac^	1 ^c^	54	2	146.5 ± 82.7

^a^: Compared with 40 mg/kg AN group, *p* < 0.05. ^b^: Compared with DCE + 40 mg/kg AN group, *p* < 0.05.^c^: Compared with fasting + 40 mg/kg AN group, *p* < 0.05; -: not detectable.

**Table 3:** Effects of fasting and DCE on tissue cyanide (CN^-^) concentrations and GSH in mice treated with AN.

Group	Brain CN^-^ μg/g Tissue	Liver CN^-^ μg/g Tissue	Liver GSH nmol/mg Prot	Brain GSH nmol/mg Prot
Normal	-	-	15.25 ± 1.99	6.22 ± 0.63
Fasting	-	-	6.83 ± 1.21 *	7.38 ± 0.06
DCE	-	-	17.48 ± 1.01	7.22 ± 0.31
Fasting + DCE	-	-	5.20 ± 1.02 *	7.27 ± 0.72
40 mg/kg AN	0.159 ± 0.038	0.607 ± 0.047	7.13 ± 2.75 *	8.01 ± 0.54
DCE + 40 mg/kg AN	-	0.024 ± 0.010 ^a^	3.33 ± 1.02 *^,#,a^	7.03 ± 0.86
Fasting + 40 mg/kg AN	0.415 ± 0.084 ^a^	0.725 ± 0.096 ^a,b^	2.51 ± 0.52 *^,#,a^	7.02 ± 0.92
Fasting + DCE + 40 mg/kg AN	0.115 ± 0.020 ^b^	0.265 ± 0.069 ^a,b,c^	2.18 ± 0.71 *^,#,a^	6.19 ± 0.51 ^a^

*: Compared with the normal group, *p* < 0.05. #: Compared with fasting group, *p* < 0.05. ^a^: Compared with 40 mg/kg AN group, *p* < 0.05. ^b^: Compared with DCE + 40 mg/kg AN group, *p* < 0.05. ^c^: Compared with fasting + 40 mg/kg AN group, *p* < 0.05; -: not detectable.; Prot: protein

## Data Availability

Not applicable.

## Abbreviations

AN	Acrylonitrile
CYP2E1	Cytochrome P450 2E1
CN^-^	Cyanide ions
DCE	trans-1,2-dichloroethylene
GSH	Glutathione
HSC	Hematopoietic stem cells
i.p.	Intraperitoneally
